# Functional Characterization of OasiCSP12: A Chemosensory Protein Regulating Olfaction and Phase Change in *Oedaleus decorus asiaticus*

**DOI:** 10.3390/insects17030256

**Published:** 2026-02-28

**Authors:** Shu Xu, Wenchang Duan, Huijuan Shi, Yajing Cai, Yaojie Zheng, Haibin Han, Ling Li, Yanyan Li, Yu Zhang

**Affiliations:** 1 Research Center for Grassland Entomology, Inner Mongolia Agricultural University, Hohhot 010020, China; xushu0904@163.com (S.X.); kyy_dwc@126.com (W.D.); 15894912121@163.com (H.S.); caiyajing112@163.com (Y.C.); zhengyaojie6100@163.com (Y.Z.); hanhaibin@imau.edu.cn (H.H.); lling@imau.edu.cn (L.L.); 2 Key Laboratory of Biohazard Monitoring, Green Prevention and Control for Artificial Grassland, Ministry of Agriculture and Rural Affairs, Institute of Grassland Research of Chinese Academy of Agricultural Sciences, Hohhot 010010, China

**Keywords:** chemosensory protein, ligand binding, molecular dynamics, *Oedaleus decorus asiaticus*, phase change

## Abstract

The Asian migratory locust (*Oedaleus decorus asiaticus*) is a major grassland pest in northern China, with outbreak dynamics closely linked to phase transition mediated by chemical communication. This study focused on a chemosensory protein, OasiCSP12, to explore its potential role in this process. We analyzed its expression patterns via qRT-PCR, purified the recombinant protein, and identified potential ligands through fluorescence competitive binding assays. Structural insights were gained through homology modeling, molecular docking, and molecular dynamics simulations, with binding energetics assessed using MM/PBSA. Results showed that *OasiCSP12* expression is phase- and sex-specific, being significantly upregulated in gregarious adult antennae. The protein bound selectively to 15 locust body-surface volatiles, including aldehydes and esters. Its structure features a hydrophobic binding cavity where van der Waals interactions, primarily predicted to be mediated by residues Val86, Leu71, and Trp101, likely stabilize ligand complexes. These findings indicate that OasiCSP12 is potentially associated with both chemical perception and phase regulation in *O. d. asiaticus*, providing a candidate target for developing behavior-based green control strategies against this pest.

## Simple Summary

*Oedaleus decorus asiaticus* is a major grassland pest whose outbreaks are driven by density-dependent phase change, a process mediated through chemical communication. This study suggests that a chemosensory protein, OasiCSP12, may participate in this process. OasiCSP12 shows phase- and sex-specific expression, with predominant levels in the antennae of gregarious adults. The protein binds specifically to 15 locust body-surface volatiles, particularly aldehydes such as heptanal. Structural analysis identified a hydrophobic binding cavity where residues Val86 and Leu71 are predicted to stabilize ligands via van der Waals interactions. These findings suggest that OasiCSP12 is a potential molecular component in both chemical perception and phase regulation, offering a possible target for the development of behavior-based, eco-friendly control strategies against locust plagues.

## 1. Introduction

The Asian migratory locust, *Oedaleus decorus asiaticus*, is a primary pest species in the arid and semi-arid grassland ecosystems of northern China, predominantly distributed in pastoral regions such as Inner Mongolia, Qinghai, and Xinjiang [1]. Both nymphs and adults feed voraciously on dominant grass species, including *Leymus chinensis* and *Stipa* spp. This sustained herbivory reduces vegetation cover and grassland productivity, potentially leading to localised grassland degradation, posing a significant threat to sustainable livestock production and ecological balance [2,3]. Outbreak years result in substantial forage losses, while factors such as overgrazing may contribute to an expansion in its range and the severity of its impact. Current management relies heavily on chemical pesticides. However, prolonged use not only risks fostering pesticide resistance but also causes secondary environmental issues, such as soil contamination and harm to non-target organisms [4,5]. Therefore, a comprehensive elucidation of the integrated mechanism governing the “expression regulation, structural characteristics, and ligand binding” of its chemosensory proteins (CSPs) could offer new insights into developing novel, environmentally friendly control strategies targeting behavioural interference, particularly phase change.

Insects depend on highly sensitive chemosensory systems to detect environmental volatile cues, regulating essential behaviours including host location, mate finding, and oviposition site selection [6,7]. Within this system, soluble chemosensory proteins (CSPs) act as small, carrier molecules featuring a hydrophobic binding pocket. They are responsible for capturing hydrophobic chemical ligands in the environment and transporting them to olfactory receptor neurons, thereby initiating downstream neuronal signalling [8,9]. The CSP family typically consists of 100–120 amino acids. Their tertiary structure is stabilised by two disulfide bridges formed by four conserved cysteine residues, folding into a compact, globular shape dominated by α-helices. These helices, along with intervening loops, form the hydrophobic binding cavity, whose geometry, hydrophobicity, and electrostatic potential determine ligand-binding specificity and affinity [10,11]. For instance, SlCSP8 from *Spodoptera litura* specifically binds the plant secondary metabolite rhodojaponin-III via hydrophobic and hydrogen-bond interactions [12], while PxylCSP18 from *Plutella xylostella* utilizes key residues such as Val30 and Leu27 to recognize various plant volatiles, participating in host selection and oviposition avoidance [6,13]. These findings provide a structural foundation for understanding CSP ligand recognition mechanisms.

Recent research has revealed that CSP functions extend beyond traditional olfaction to include roles in insecticide resistance and environmental stress response [2,14,15]. In the brown planthopper (*Nilaparvata lugens*), biotin deficiency activates the ROS/CncC-AhR/ARNT signalling pathway, upregulating genes such as *CSP2* and *CSP4*. These CSPs can bind the insecticide imidacloprid with high affinity, sequestering it extracellularly and reducing its effective concentration, thereby contributing to chemical resistance [16]. Similarly, AgosCSP5 in the cotton aphid (*Aphis gossypii*) binds various insecticide molecules, forming a molecular basis for resistance evolution [10]. Furthermore, CSPs are crucial for soil-oriented behaviours in subterranean pests. For example, *CSP2* expression in larvae of *Hylamorpha elegans* correlates positively with soil organic matter content, and its silencing significantly impairs the larvae’s ability to locate high-organic-matter substrates [17]. The silkworm (*Bombyx mori*) CSP16 efficiently binds multiple plant volatiles, hinting at a role in host recognition [4]. These advances underscore the central role of CSPs in helping insects adapt to complex chemical ecologies and provide a theoretical basis for considering them as potential targets for pest control.

In locust chemosensory research, emerging evidence suggests the involvement of CSPs in communication related to phase change. Transcriptomic analysis of *O. d. asiaticus* revealed differential expression of several CSP genes, including *OasiCSP12*, between the gregarious and solitary phases. Notably, *OasiCSP12* expression was significantly higher in the antennae of gregarious adults, suggesting a potential role in the recognition and transduction of chemical signals during phase change. Despite these insights, significant gaps remain in our understanding of CSPs in *O. d. asiaticus*: (i) the binding specificity of this protein for locust body surface volatiles, its three-dimensional structural features, and the underlying dynamic interaction mechanisms remain elusive; and (ii) the rational design of behaviour-disrupting agents targeting CSPs lacks a robust structural biology foundation.

Therefore, this study focuses on “phase-change behavioural regulation” and aims to systematically elucidate the dual functions of OasiCSP12 in both chemoreception and phase transition in *O. d. asiaticus*. The specific objectives are: (1) to analyse the expression patterns of *OasiCSP12* across various tissues in both gregarious and solitary locusts, as well as its dynamic shifts during phase transition; (2) to establish a prokaryotic expression and purification protocol to obtain active recombinant OasiCSP12 protein; (3) to screen for natural ligands via fluorescence competitive binding assays and characterize the 3D structure and protein-ligand interaction through integrated homology modelling, molecular docking, and 200 ns molecular dynamics simulations; and (4) to employ the MM/PBSA method to calculate binding free energies and identify key contributing residues. The findings are expected to provide novel insights into the molecular mechanisms governing phase change and chemoreception in *O. d. asiaticus*, laying a theoretical foundation for developing a dual-mode green control strategies that combine “phase-change interference” with “olfactory disruption.”

## 2. Materials and Methods

### 2.1. Insect Rearing and Sample Preparation

*Oedaleus decorus asiaticus* were collected from the Xianghuangqi grassland in Xilingol League, Inner Mongolia, in late June 2024. In the laboratory, locusts were preliminarily categorized by body color and sex. They were subsequently reared at two distinct population densities to induce gregarious (200 individuals per cage) and solitary (10 individuals per cage) phases, housed in separate, well-ventilated rooms. All insects were fed fresh maize leaves and maintained under controlled conditions: temperature 27 ± 1 °C, relative humidity 50–70%, and a photoperiod of 16L:8D.

Based on preliminary experiments indicating a peak in CSP expression at 6 h post-treatment, samples were collected at this time point for tissue-specific analysis. The following eight tissue types were dissected: head (without antennae), thorax, abdomen, wings, antennae, forelegs, midlegs, and hindlegs. Each tissue sample pool consisted of material from 15 locusts, with three biological replicates prepared per tissue type. All samples were immediately frozen in liquid nitrogen and stored at −80 °C.

Total RNA was extracted using the Eastep™ Super Total RNA Extraction Kit (Promega, Beijing, China). First-strand cDNA synthesis was performed with the PrimeScript™ RT Reagent Kit with gDNA Eraser (TaKaRa, Dalian, China). Escherichia coli XL1-Blue competent cells were obtained from Stratagene (La Jolla, CA, USA), and Coomassie Brilliant Blue R-250 was purchased from Huazhong Haiwei (Beijing, China) Gene Technology Co., Ltd. Key instruments included a high-speed refrigerated centrifuge (5415D, Eppendorf, Hamburg, Germany), a NanoDrop spectrophotometer (Thermo Fisher Scientific, Waltham, MA, USA), a real-time quantitative PCR system (FTC-3000, Funglyn Biotech, Richmond Hill, ON, Canada), and a thermal cycler (MyCycler, Bio-Rad, Hercules, CA, USA).

### 2.2. Experimental Procedures

#### 2.2.1. Phase Change Treatment of Locusts

Solitarization of Gregarious Locusts: Gregarious-phase adults were transferred to individual boxes at a low density of 10 individuals per box. Samples were collected at 0, 1, 6, 12, 24, and 72 h post-transfer, with three replicates (15 locusts each) per time point.

Gregarization of Solitary Locusts: Solitary-phase adults were subjected to crowding in cages at a high density of 200 individuals per cage. Samples were collected at the same time intervals as above, with three replicates per time point.

Sampling and subsequent handling followed the protocol described in Section 2.1.

#### 2.2.2. Quantitative Real-Time PCR (qRT-PCR)

Gene-specific primers for *OasiCSP12* were designed using Primer3 online software (v.3.3.0, https://www.primer3plus.com, accessed on 20 June 2024), with β-actin serving as the internal reference gene. Primers were synthesized by Sangon Biotech (Shanghai, China; sequences in Table 1). qRT-PCR was performed using MonAmp™ SYBR^®^ Green qPCR Mix (Low ROX) (Monad Biotech Co., Ltd., Suzhou, China) in a 20 µL reaction volume containing 1 µL of cDNA template, 0.4 µL each of forward and reverse primers, 10 µL of 2× SYBR Green Master Mix (Monad Biotech Co., Ltd., Suzhou, China), and 8.2 µL of nuclease-free water. Three biological and three technical replicates were performed for each sample. Expression in the male head tissue was used as a positive control. The thermal cycling protocol was: 95 °C for 10 min; 40 cycles of 95 °C for 15 s and 60 °C for 1 min; followed by a dissociation curve analysis (95 °C for 15 s, 60 °C for 15 s, 95 °C for 15 s).

Relative expression levels were calculated using the 2^−ΔΔCt^ method. Statistical analysis was conducted with IBM SPSS Statistics 26. Pairwise comparisons of *OasiCSP12* expression between sexes within the same tissue were performed using Student’s t-test. Differences in expression levels across different tissues within the same sex were analyzed by one-way ANOVA followed by Duncan’s multiple range test. A *p*-value < 0.05 was considered statistically significant.

#### 2.2.3. RNA Extraction and cDNA Synthesis

Frozen tissue samples (~40 mg) were ground to a fine powder in liquid nitrogen. Total RNA was extracted using the specified kit according to the manufacturer’s protocol, including an on-column DNase I digestion step to eliminate genomic DNA. RNA concentration and purity were assessed spectrophotometrically. First-strand cDNA was synthesized from 1 µg of total RNA using the PrimeScript™ RT reagent kit (Takara Bio Inc., Kusatsu, Japan) and stored at –20 °C.

#### 2.2.4. Gene Cloning of OasiCSP12

Specific primers flanked by *KpnI* and *HindIII* restriction sites were designed based on the known *OasiCSP12* sequence (SnapGene software; https://www.snapgene.com; accessed on 15 July 2024) to exclude the hydrophobic signal peptide. Primer sequences were: forward 5′-GGTACCATGCAGACGCCGACGC-3′ (KpnI); reverse 5′-AAGCTTTCACTGTTGCTGTTGCTGCTG-3′ (HindIII). The target fragment was amplified by RT-PCR, gel-purified, and cloned into the pMD19-T vector. After transformation into *E. coli* DH5α competent cells, positive clones were identified by colony PCR and verified by sequencing. The confirmed *OasiCSP12* fragment was subsequently subcloned into the pQE-40 expression vector via double digestion with *BamHI* and *HindIII*.

#### 2.2.5. Prokaryotic Expression and Purification of OasiCSP12 Protein

The recombinant pQE-40-OasiCSP12 plasmid was transformed into *E. coli BL21* (DE3) competent cells. A single positive colony was inoculated into LB medium containing kanamycin (100 µg/mL). Protein expression was induced at OD600≈0.6 with 1 mM isopropyl β-D-1-thiogalactopyranoside (IPTG) for 4 h at 30 °C. Bacterial cells were harvested by centrifugation, resuspended in Lysis Buffer B (50 mM NaH_2_PO_4_, 300 mM NaCl, and 5 mM imidazole, with the pH adjusted to 7.4), and disrupted by sonication on ice. The soluble fraction, obtained by centrifugation, was applied to a Ni-NTA affinity chromatography column. The His-tagged OasiCSP12 protein was eluted with imidazole-containing Elution Buffer E (50 mM NaH_2_PO_4_, 300 mM NaCl, and 300 mM imidazole, with the pH adjusted to 7.4). Protein purity and molecular weight were confirmed by 12.5% SDS-PAGE stained with Coomassie Brilliant Blue R-250.

#### 2.2.6. Fluorescence Competitive Binding Assay

The fluorescent probe 1-N-phenylnaphthylamine (1-NPN) and 21 candidate locust body-surface volatiles (identified in prior work) were dissolved in HPLC-grade methanol to prepare 1 mM stock solutions [18]. rPurified OasiCSP12 protein was diluted to 2 µM in 50 mM Tris-HCl buffer (pH 7.4). Fluorescence spectra were recorded with excitation at 337 nm (slit width 10 nm) and emission scanned from 350 to 550 nm (slit width 10 nm).

First, the binding affinity (K_1-NPN_) of 1-NPN to OasiCSP12 was determined by titrating aliquots of 1-NPN (increasing final concentration by 2 µM per step) into 2 mL of protein solution until fluorescence saturation. For competitive binding, a solution containing 2 µM OasiCSP12 and a saturating concentration of 1-NPN was prepared. Aliquots of each competitor ligand were then successively added (2 µM increments), and the decrease in fluorescence intensity was recorded. The concentration of competitor that reduced the initial fluorescence by 50% (IC_50_) was used to calculate the dissociation constant (Ki) for each ligand with the formula: Ki = [IC_50_]/(1 + [1-NPN]/K_d_). [1-NPN] represents the free concentration of 1-NPN (1.48 µmol/L), and K_d_ is the dissociation constant of 1-NPN binding to OasiCSP12.

#### 2.2.7. Tertiary Structure Prediction and Molecular Docking

The amino acid sequence of OasiCSP12 (GenBank accession: KX905068) was retrieved from NCBI. The 3D structures of the 21 candidate ligands were obtained from the PubChem database (https://pubchem.ncbi.nlm.nih.gov, accessed on 12 March 2025). The tertiary structure of OasiCSP12 was predicted using the Boltz-2 module (an AlphaFold3-like model) on the WeMol platform (https://wemol.wecomput.com/, accessed on 12 March 2025), generating five candidate models [18,19]. The model with the highest confidence score was selected, structurally optimized, and validated using the PROCHECK program via the SAVES online suite (https://saves.mbi.ucla.edu/, accessed on 20 March 2025).

Molecular docking was performed using the AutoDock-GPU v2 module on the WeMol platform. A rigid docking approach was employed to simulate the binding of OasiCSP12 with each ligand. For each protein-ligand pair, five docking poses were generated and ranked based on predicted binding affinity (log(IC_50_)). The pose with the lowest binding energy score for each ligand was selected for subsequent analysis.

#### 2.2.8. Molecular Dynamics Simulation

Fully automated MD simulations were performed using GROMACS (v.2025.0; https://www.gromacs.org, accessed on 20 March 2025) via the WeMol platform. Initially, the starting PDB file was repaired, and the protein, ligand, and nucleic acid components were separated. Protein residue pKa values were predicted at pH 7.0 (with charged N- and C-terminal) to generate a protonated protein PDB file. The AMBER03 force field and SPC water model were selected to construct the receptor topology file. After assigning charges to the ligand using the bond charge correction (BCC) method, the protein-ligand complex was solvated in a water box. Na^+^/Cl^−^ ions were added to neutralize the system charge, and periodic boundary conditions (PBC) were applied in all three dimensions.

Energy minimization was conducted using the steepest descent algorithm (maximum force < 100 kJ/(mol·nm)), with long-range electrostatic interactions calculated using the Particle Mesh Ewald (PME) method and short-range non-bonded interactions handled with a cutoff scheme (cutoff distance of 1.2 nm). Subsequently, the system was equilibrated: first under the NVT ensemble (300 K, temperature coupled using the V-rescale method, 0.2 ps), followed by equilibration under the NPT ensemble (1 bar, pressure coupled using the Berendsen method, 2 ps), with an integration step size of 0.001 ps for both equilibration phases. Finally, a 200 ns production simulation was performed under the NPT ensemble using the leap-frog integrator with a 0.001 ps step size. The resulting trajectory was used for RMSD and RMSF analyses to evaluate the stability and conformational changes in the protein-ligand complex.

#### 2.2.9. Binding Free Energy Calculation

This study employed the Molecular Mechanics/Poisson-Boltzmann Surface Area (MMPBSA) method to calculate the binding free energy between the receptor and ligand, while simultaneously obtaining energy decomposition data, the association constant (Ka), and the inhibitor constant (*Ki*). The binding free energy comprises the ensemble average of the receptor–ligand interaction energy (<ΔEplint>), the solvation free energy (ΔGsol = ΔGpb + ΔGnp), and the entropy contribution (−TΔS). The −TΔS term was computed using the interaction entropy method, expressed as −TΔS_IE_ = k_B_TIn <exp(βΔEplint)>, where k_B_ is the Boltzmann constant, β = 1/(k_B_T), ΔEplint = Eplint − <Eplint>, and <·> denotes the ensemble average. The calculations were performed using structurally stable segments from the molecular dynamics (MD) trajectories, as validated by root-mean-square deviation (RMSD) analysis [20]. Two computational modes were adopted: the Trajectory mode, which extracts conformations from the stable portion of the MD trajectory for calculation; and the One-Structure mode, which inputs a PDB structure (either crystal or docked), performs energy minimization, and then proceeds with the calculation. Ka was derived from the binding free energy, while Ki was determined based on the computed binding energetics to complement the experimental data.

## 3. Results

### 3.1. Phase- and Sex-Specific Expression Patterns of OasiCSP12

**Figure 1:**
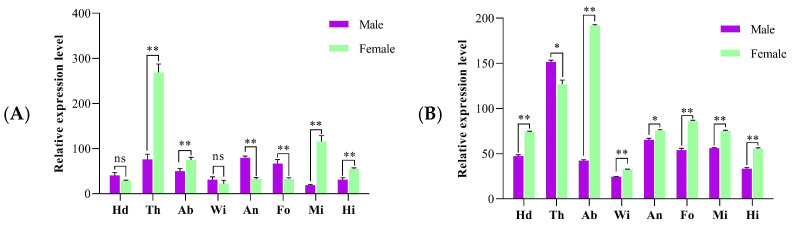
Expression profiles of *OasiCSP12* in different tissues of adult male and female solitary and gregarious *O. d. asiaticus*. (**A**) Expression profiles in solitary locusts; (**B**) Expression profiles in gregarious locusts. Relative expression levels were normalized to the *OasiCSP12* expression level in the heads (without antennae) of adult males as the control. Asterisks above the columns represent significant differences between the same tissues of females and males (**: *p* < 0.01; *: *p* < 0.05; ns: no significant difference). Hd: head (without antennae), Th: thorax, Ab: abdomen, Wi: wings, An: antennae, Fo: forelegs, Mi: midlegs, Hi: hindlegs.

Quantitative real-time PCR (qRT-PCR) analysis revealed distinct tissue-specific and sex-dependent expression profiles for *OasiCSP12* in *O. d. asiaticus*. In solitary-phase locusts, *OasiCSP12* expression was significantly higher in the thorax of both sexes compared to other tissues. In the solitary phase, the relative expression levels of *OasiCSP12* in female tissues were markedly higher than those in males, with female thoracic expression being 3.53-fold greater than that in males, and female antennal expression 3.37-fold higher (Figure 1A).

In gregarious-phase locusts, the highest expression was observed in the abdomen of females and the thorax of males. Overall, expression levels across various tissues were generally higher in females than in males; for instance, female abdominal expression was 1.27-fold that of male thoracic expression. Besides the thorax and abdomen, *OasiCSP12* was also expressed at considerable levels in antennae, suggesting its potential involvement in both chemosensory signal recognition and non-chemosensory physiological processes (Figure 1B).

### 3.2. Dynamic Expression of OasiCSP12 During Phase Change

The expression dynamics of OasiCSP12 during forced phase transition were investigated. During the gregarization of solitary locusts (low-to-high density), *OasiCSP12* expression in male antennae showed a gradual increasing trend, becoming significantly elevated after 24 h. In contrast, female antennal expression exhibited minor fluctuations without a clear time-dependent pattern.

**Figure 2:**
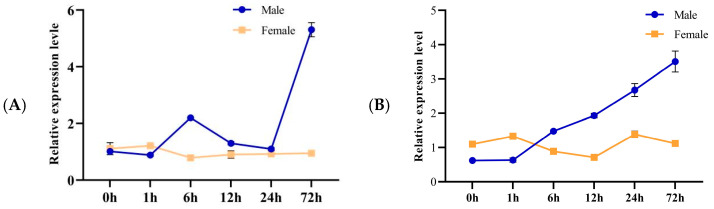
Expression dynamics of *OasiCSP12* in antennae during solitary-to-gregarious and gregarious-to-solitary phase transitions of *O. d. asiaticus*. (**A**): Expression trend of *OasiCSP12* in antennae during the solitary-to-gregarious phase transition of *O. d. asiaticus*; (**B**): Expression trend of *OasiCSP12* in antennae during the gregarious-to-solitary phase transition of *O. d. asiaticus*.

During the gregarious transition of solitary locusts (low-to-high density), the relative expression level of *OasiCSP12* in male antennae showed significant fluctuations: an increasing trend from 0 to 6 h, a slight decline from 6 to 12 h, a sharp increase after 24 h, and peaking at 72 h. In contrast, the relative expression level in females remained consistently low throughout the time course, with no significant fluctuations among time points (Figure 2A). During the solitarization of gregarious locusts (high-to-low density), the relative expression level of *OasiCSP12* in male antennae exhibited a gradual upward trend with prolonged treatment time, and increased significantly after 24 h. Although the relative expression level in females showed minor fluctuations, no obvious time-dependent changes were observed. Under both phase transition treatments, significant differences in *OasiCSP12* relative expression were detected between males and females, indicating that the transcriptional regulation of this gene is sex-specific, and its dynamic changes are closely associated with the phase change process of *O. d. asiaticus* (Figure 2B).

### 3.3. Prokaryotic Expression and Purification of Recombinant OasiCSP12

**Figure 3:**
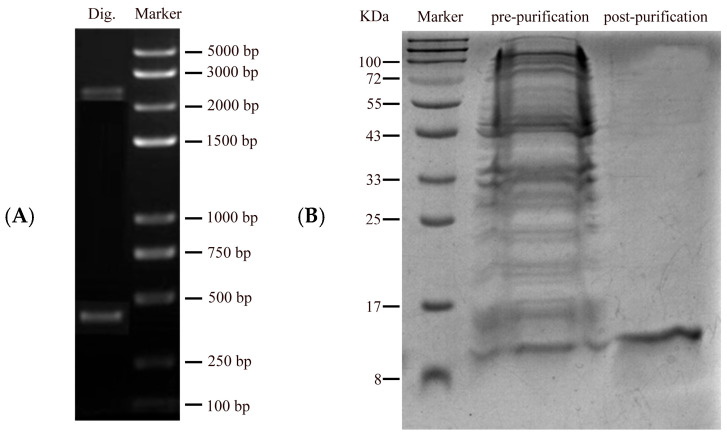
Electrophoretic analysis of restriction enzyme digestion identification, protein expression and purification of OasiCSP12 (Dig., restriction enzyme digestion product). (**A**) Restriction enzyme digestion identification of OasiCSP12; (**B**) Electrophoretic analysis of protein expression and purification of OasiCSP12.

The OasiCSP12 gene was successfully cloned and expressed in E. coli. Double digestion of the recombinant pQE40-OasiCSP12 plasmid yielded two clear DNA fragments, confirming correct vector construction (Figure 3A). Following IPTG induction and purification via Ni-NTA affinity chromatography, SDS-PAGE analysis showed a single predominant protein band at approximately 15 kDa (Figure 3B), consistent with the predicted molecular mass of OasiCSP12 (15.03 kDa). The concentration of the purified protein was determined to be 0.83 mg/mL (68.24 µM) using the BCA method, which was sufficient for subsequent experiments.

### 3.4. Ligand-Binding Specificity of OasiCSP12

**Figure 4:**
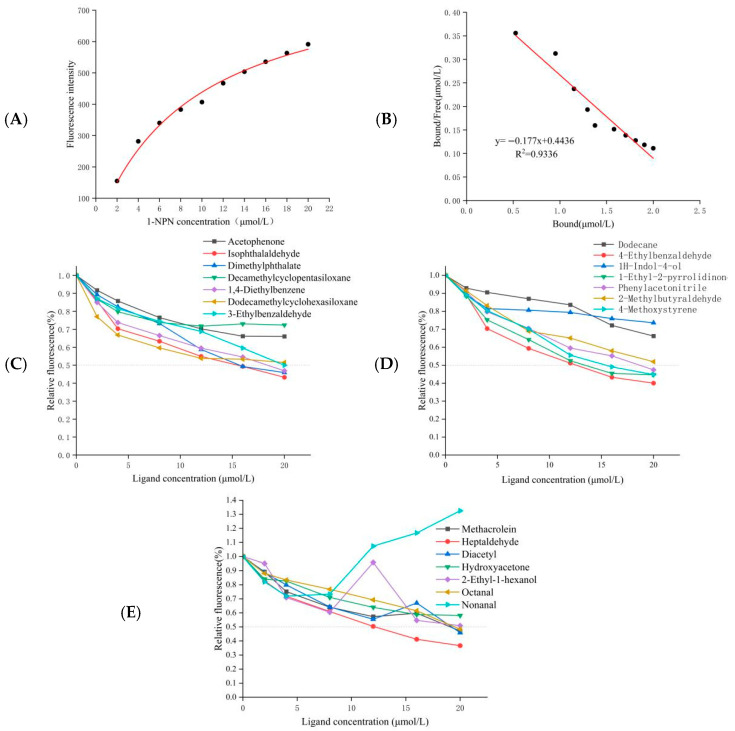
Fluorescence competitive binding assays of OasiCSP12 Protein. (**A**) Binding curves of recombinant OasiCSP12 protein with the fluorescent probe 1-NPN in pH 7.4 buffer system; (**B**) Scatchard equation analysis curve; (**C**–**E**) Competitive binding curves of recombinant OasiCSP12 protein with 21 cuticular volatiles from *O. d. asiaticus*.

A fluorescence competitive binding assay was employed to characterize the ligand-binding profile of OasiCSP12. Saturation binding experiments using the fluorescent probe 1-NPN yielded a dissociation constant (K_d_) of 9.097 µM, confirming a good binding affinity between OasiCSP12 and 1-NPN (Figure 4A,B).

Subsequent competitive binding assays with 21 candidate locust body-surface volatiles identified 15 compounds that competitively displaced 1-NPN, indicating specific binding to OasiCSP12 (Ki < 30 µM) (Figure 4C–E; Table 2). These ligands included aldehydes (e.g., heptanal, 2-methylbutanal, octanal), esters (e.g., dimethyl phthalate), and nitrogen-containing compounds (e.g., benzonitrile, 1-ethyl-2-pyrrolidinone). Heptanal exhibited the highest affinity (Ki < 10 µM). Six compounds, including decamethylcyclopentasiloxane and acetophenone, showed no significant binding (Ki > 30 µM).

### 3.5. Predicted Tertiary Structure and Molecular Docking Analysis

**Figure 5:**
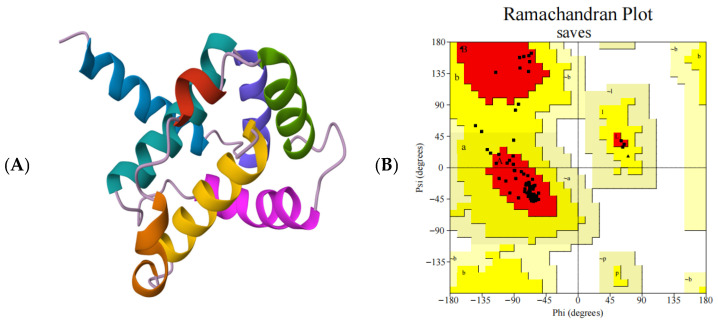

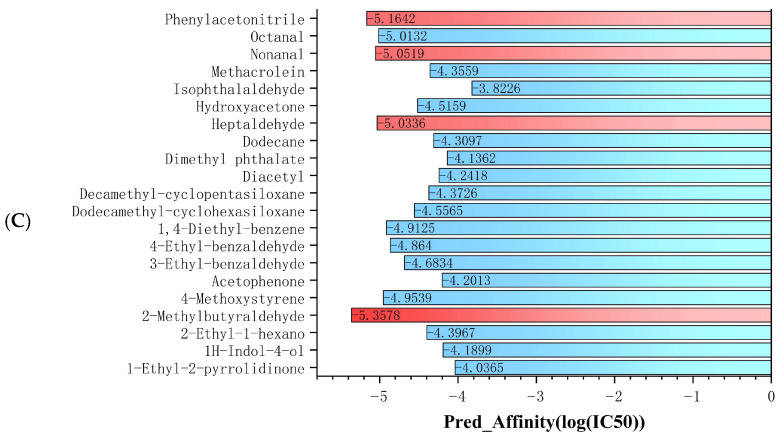
Structural modeling, conformational rationality verification and ligand binding affinity analysis of OasiCSP12. (**A**) Tertiary structural model of OasiCSP12 protein; (**B**) Ramachandran plot of OasiCSP12 (results of ProCheck verification, where red, brown, and yellow regions represent the most favored, additionally allowed, and generously allowed regions, respectively); (**C**) Predicted binding affinities of 21 ligands with OasiCSP12 from molecular docking (Pred_Affinity(log(IC_50_))). The four ligands with the highest predicted binding affinities are highlighted in red.

The tertiary structure of OasiCSP12 was predicted using homology modeling. The model features a typical CSP fold, where α-helices and intervening loops form a hydrophobic binding pocket (Figure 5A). Ramachandran plot analysis via PROCHECK indicated that 93.4% of residues were in the most favored regions, and 6.6% in additional allowed regions, validating the model’s stereochemical quality (Figure 5B).

The binding affinities of 21 different types of ligands to OasiCSP12 were predicted and analyzed via molecular docking (Figure 5C). The results showed that the predicted binding affinities (Pred_Affinity (log(IC_50_))) of the ligands to OasiCSP12 ranged from −3.8226 to −5.3578. Among these, aldehydes exhibited the most prominent binding capabilities: 2-methylbutanal had the highest binding affinity (log(IC_50_) = −5.3578), while nonanal (−5.0519), heptanal (−5.0336), and octanal (−5.0132) also displayed strong binding potential in molecular docking (all log(IC_50_) values below −5.0). In contrast, heterocyclic compounds or aromatic dialdehyde ligands such as isophthalaldehyde (−3.8226) and 1-ethyl-2-pyrrolidone (−4.0365) showed relatively weak binding affinities.

### 3.6. Molecular Dynamics Simulations and Binding Free Energy Calculations

#### 3.6.1. Complex Stability

To establish a gradient comparison based on fluorescence competitive binding results and optimize molecular dynamics (MD) simulation design, four representative ligands were selected for MD analysis. Heptanal exhibited the strongest binding activity (Ki = 9.72 μM), while 2-methylbutanal (Ki = 21.57 μM) and benzonitrile (Ki = 17.19 μM) showed moderate activity. In contrast, nonanal displayed no binding activity (Ki > 30 μM) in the functional assay, representing the weakest binding among the selected candidates. MD simulation revealed distinct differences in conformational stability among these OasiCSP12-ligand complexes over the 200 ns simulation.

**Figure 6:**
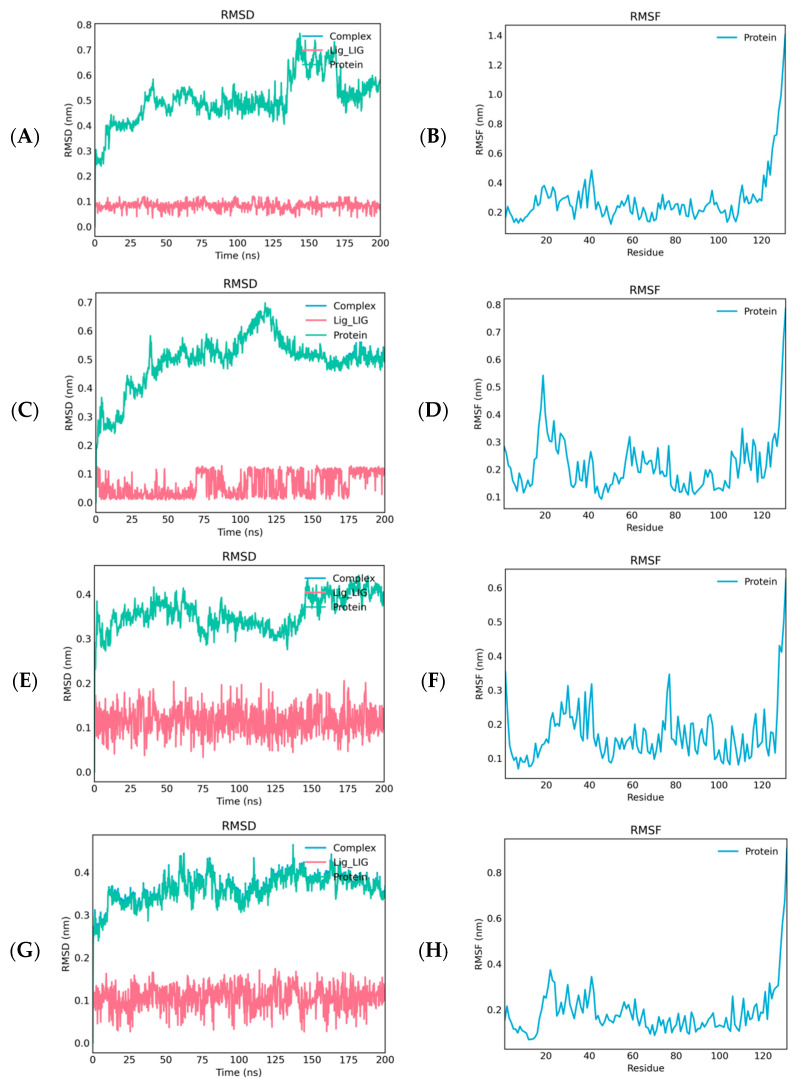
RMSD and RMSF analysis of molecular dynamics simulations for OasiCSP12 with 2-methylbutanal, phenylacetonitrile and nonanal. (**A**) RMSD variation of 2-Methylbutanal; (**B**) RMSF of OasiCSP12 in the 2-methylbutanal system; (**C**) RMSD variation in phenylacetonitrile; (**D**) RMSF of OasiCSP12 in phenylacetonitrile system; (**E**) RMSD variation in nonanal; (**F**) RMSF of OasiCSP12 in the nonanal system; (**G**) RMSD variation in heptanal; (**H**) RMSF of OasiCSP12 in heptanal system.

The root-mean-square deviation (RMSD) was employed to evaluate the conformational fluctuations of the complexes. The OasiCSP12-heptanal complex exhibited the smallest RMSD fluctuations, stabilizing at 0.3–0.4 nm in the late stage, with the ligand’s RMSD consistently remaining below 0.1 nm, indicating optimal binding stability. The OasiCSP12-2-methylbutanal complex showed minor fluctuations, stabilizing at approximately 0.4 nm, with the ligand RMSD ranging between 0.1 and 0.2 nm. Similarly, the OasiCSP12-benzonitrile complex reached equilibrium between 0.5 and 0.6 nm, with the ligand remaining stable at 0.0–0.1 nm. Notably, the OasiCSP12-nonanal complex’s RMSD increased significantly after 150 ns, peaking above 0.7 nm; while the ligand itself remained at ~0.1 nm, the protein backbone exhibited substantial late-stage fluctuations, suggesting an unstable binding environment (Figure 6A,C,E,G).

Root-mean-square fluctuation (RMSF) analysis characterized the residue flexibility of OasiCSP12. Across all four complexes, most OasiCSP12 residues had RMSF values between 0.1 and 0.5 nm, with minimal conformational fluctuations in the core region. Only the C-terminal region (after residue ~120) showed a marked increase in RMSF (up to 0.7–1.4 nm), indicating high flexibility in this region. Different ligands differentially affected OasiCSP12 residue flexibility: binding to heptanal resulted in the lowest overall RMSF values and the weakest flexibility in the core region; binding to 2-methylbutanal or benzonitrile led to intermediate flexibility between the heptanal and nonanal complexes; binding to nonanal caused the largest residue fluctuations in specific protein regions (Figure 6B,D,F,H). Collectively, the OasiCSP12-heptanal complex exhibited the highest conformational stability, followed by the 2-methylbutanal and benzonitrile complexes. The significant late-stage fluctuations observed in the nonanal complex mirrored the lack of activity observed in the fluorescence competitive binding assay, thereby validating the reliability of our integrated experimental and computational approach.

In the initial stage, the RMSD values of OasiCSP12 and all four ligands fluctuated considerably. After 150 ns, both the protein and ligands converged to approximately 0.3–0.5 nm, indicating that the conformations reached equilibrium and the binding between the protein and ligands was stable.

#### 3.6.2. Binding Free Energy and Key Residues

Based on the molecular dynamics simulation results, the stable conformational stage from 180 to 200 ns was selected. Binding free energies and residue contributions were calculated using the MM/PBSA method to clarify the binding site characteristics and interaction mechanisms of the four ligands with OasiCSP12 (Table 3). The total binding free energies of the four ligands to OasiCSP12 were −30.283 kcal/mol (2-methylbutanal), −34.144 kcal/mol (benzonitrile), −49.957 kcal/mol (nonanal), and −44.509 kcal/mol (heptanal), respectively. The binding affinities ranked from strongest to weakest as follows: nonanal > heptanal > benzonitrile > 2-methylbutanal. Notably, nonanal exhibited the highest binding affinity to OasiCSP12; excluding nonanal, the remaining results were generally consistent with those of the fluorescence competitive binding assay.

The core residues contributing to the binding of 2-methylbutanal to OasiCSP12, ordered by contribution strength, were VAL86 (−3.527 kcal/mol), LEU71 (−3.116 kcal/mol), LEU8 (−2.284 kcal/mol), VAL90 (−2.137 kcal/mol), and TRP101 (−1.776 kcal/mol). For benzonitrile binding to OasiCSP12, the core residues were LEU71 (−7.271 kcal/mol), ILE118 (−5.015 kcal/mol), VAL90 (−3.420 kcal/mol), TRP101 (−2.564 kcal/mol), and TYR108 (−2.321 kcal/mol). The core residues for nonanal binding to OasiCSP12 were LEU63 (−4.983 kcal/mol), ILE60 (−4.157 kcal/mol), TYR28 (−3.690 kcal/mol), VAL89 (−3.085 kcal/mol), and VAL33 (−2.623 kcal/mol). For heptanal binding to OasiCSP12, the core residues were ILE118 (−4.620 kcal/mol), LEU104 (−4.512 kcal/mol), TRP101 (−4.418 kcal/mol), LEU71 (−3.595 kcal/mol), and LEU15 (−2.593 kcal/mol).

Binding of all four ligands to OasiCSP12 was dominated by Van der Waals forces. Nonanal achieved binding through the high matching degree between its long hydrocarbon chain and the hydrophobic pocket, albeit with large conformational fluctuations. Heptanal formed stable binding via precise interactions with core hydrophobic residues. 2-methylbutanal exhibited an “efficient and stable” binding mode characterized by concentrated contributions from core residues and a few inhibitory residues. Benzonitrile relied on aromatic ring π-π stacking and strong polar interactions, resulting in a more complex binding site architecture.

## 4. Discussion

Chemosensory proteins (CSPs) are thought to be pivotal in insect physiological adaptability, serving as carriers for hydrophobic molecules that regulate diverse behaviors [21,22]. In this study, we functionally characterized *OasiCSP12* from *Oedaleus decorus asiaticus*. Our results suggest that *OasiCSP12* exhibits a distinct, density-dependent expression profile and possesses high binding affinity for specific host-plant volatiles and aggregation pheromones. These findings provide preliminary support for the hypothesis that *OasiCSP12* may act as a molecular bridge linking olfactory perception to the physiological regulation of phase polyphenism.

Our transcriptional analysis reveals that *OasiCSP12* expression is characterized by marked sex- and phase-specificity. In solitary locusts, expression was predominantly thoracic, with females exhibiting significantly higher levels than males. Conversely, in the gregarious phase, the highest expression in females shifted to the abdomen, whereas male expression remained thorax-dominant. This differential pattern mirrors that of PxylCSP18 in the diamondback moth [6], suggesting that OasiCSP12 might facilitate sex-specific behaviors such as oviposition site selection and mate recognition. Furthermore, the consistently high expression in antennae across phases underscores its core function in olfactory signal transduction [23].

The dynamic regulation of OasiCSP12 during forced phase transition points to its potential involvement in the gregarious-solitary shift. During crowding (solitary-to-gregarious), male antennal expression upregulated significantly after 24 h. In contrast, during isolation (gregarious-to-solitary), male expression exhibited a fluctuating “increase-decline-rise” pattern, peaking at 72 h. This sex-specific plasticity aligns with mechanisms reported for OasiCSP4 [24], suggesting that OasiCSP12 may participate in the early perception of density signals. The observed transcriptional peaks at 6 or 24 h are consistent with the rapid response characteristics of CSP genes to environmental stimuli [25], could offer potential molecular markers for monitoring the early stages of phase change.

Functionally, OasiCSP12 demonstrates selective ligand binding. Fluorescence competitive binding assays identified 15 locust body-surface volatiles as potential ligands (Ki < 30 µM), with a distinct preference for aldehydes such as heptanal (Ki < 10 µM) and 2-methylbutanal [26]. This affinity for aldehydes/alcohols parallels the profile of LmigOBP1 in the migratory locust [27], suggesting that these compounds might be conserved semiochemicals for acridid CSPs.

The predicted tertiary structure of OasiCSP12 features a hydrophobic binding pocket formed by α-helices and random coils, a hallmark of insect CSPs [28,29]. Model validation via PROCHECK indicated excellent stereochemical quality, with 93.4% of residues in the most favored regions of the Ramachandran plot [30], providing a reliable structural framework. Molecular docking predicted the highest binding affinity for 2-methylbutanal (predicted log(IC_50_) = −5.3578), where the aldehyde group is suggested to form interactions with potential key residues like Val86 and Leu71. Slightly weaker predicted affinities for ligands like nonanal and heptanal might relate to differences in carbon chain length and optimal cavity fit [31].

Notably, the C-terminal region (beyond approximately residue 120) displayed high flexibility in MD simulations, with RMSF values reaching 0.7–1.4 nm. This dynamic feature is reminiscent of SgreCSP4 in the desert locust [32] and may be functionally relevant. A flexible C-terminus might undergo conformational adjustments to partially “wrap” the bound ligand, shielding it from the aqueous environment and potentially enhancing binding stability [22,33]. The pronounced hydrophobicity of the binding cavity appears to favor lipophilic ligands like aldehydes and aromatic compounds [34], potentially adapting OasiCSP12 to recognize the predominantly hydrophobic volatiles found on the locust cuticle.

This study integrated experimental and computational approaches to evaluate ligand specificity. While molecular docking predicted high affinities for several compounds, subsequent 200 ns MD simulations revealed crucial dynamic distinctions. Specifically, the OasiCSP12-heptanal complex maintained superior conformational stability with minimal RMSD fluctuations, whereas nonanal and benzonitrile exhibited pronounced RMSF fluctuations, indicating potential structural instability. This dynamic instability provides a plausible explanation for the discrepancy observed in fluorescence assays, where nonanal failed to exhibit competitive binding despite its favorable docking score. Therefore, MD simulations served as an indispensable diagnostic tool to identify nonanal as a “false positive” from static screening, highlighting that dynamic stability might be a key determinant of ligand affinity.

The failure of certain predicted ligands to displace the 1-NPN probe in vitro likely also stems from the inherent limitations of surrogate markers and experimental conditions. As a synthetic probe, 1-NPN possesses a distinct aromatic structure that may occupy secondary binding sites or regions only partially overlapping with natural volatiles, potentially leading to atypical binding modes [35]. Furthermore, while MD simulations provide a high-resolution view of protein-ligand dynamics, they cannot fully replicate the multifaceted experimental environment—such as variations in pH, ionic strength, and buffer composition—which significantly modulate protein flexibility and ligand solubility [36]. These findings underscore that a more complete understanding of OasiCSP12’s recognition properties requires the complementary use of both experimental assays and dynamic simulations to bridge the gap between in silico models and physiological reality.

The ligand selection mechanism of OasiCSP12 is proposed to involve a dual-filter process: primary steric screening by cavity size (accommodating C5–C9 aldehydes) and secondary chemical complementarity potentially driven by van der Waals interactions with key residues like Val86 and Leu71. This “spatial fit plus chemical complementarity” logic parallels sex pheromone recognition by MsepPBP3 [37].

Ecologically, the functional attributes of OasiCSP12 suggest possible behavioral implications. First, its phase-specific expression and affinity for body-surface volatiles imply a potential role in the “chemoreception-phase response” pathway, modulating intraspecific communication [24,38]. Second, its binding to 2-methylbutanal suggests a possible involvement in detecting aggregation pheromone precursors within gregarious bands [39]. Third, the strong affinity for heptanal (a key volatile of maize and wheat) points to a possible function in host location [40,41].

It is important to emphasize that this study has several limitations. The proposed functions of key residues (Val86, Leu71) are solely based on computational simulations and lack direct validation via site-directed mutagenesis. Additionally, the observed link between expression dynamics and behavioral output remains strictly correlative at this stage. Our in silico and in vitro results should be interpreted as predictive rather than definitive proof of in vivo activity. Future research employing RNA interference (RNAi) or CRISPR/Cas-9 gene editing, combined with behavioral bioassays, will be required to unequivocally establish the regulatory mechanisms and biological functions of OasiCSP12.

## 5. Conclusions

This study provides a comprehensive functional and structural characterization of the chemosensory protein *OasiCSP12* in the Asian migratory locust, *O. d. asiaticus*. We demonstrated that *OasiCSP12* exhibits distinct, phase- and sex-specific expression patterns, characterized by dynamic regulation during density-driven phase transitions, particularly in male antennae. The recombinant OasiCSP12 protein was successfully expressed and purified, enabling the identification of 15 locust body-surface volatiles as potential binding ligands, with a notable preference for aldehydes such as heptanal. Integrated structural modeling and molecular dynamics simulations suggest a canonical hydrophobic binding cavity and point to the possible involvement of residues Val86 and Leu71 in ligand stabilization, primarily via van der Waals interactions. These findings suggest a potential association between the molecular properties of *OasiCSP12* and the chemical ecology of *O. d. asiaticus*, indicating that it may participate in both chemical perception and phase change regulation. While further in vivo studies are needed to confirm these biological roles, our findings advance the understanding of the chemosensory mechanisms underlying locust phase polyphenism and identify *OasiCSP12* as a candidate molecular target for the development of novel, behavior-based green control strategies.

## Figures and Tables

**Table 1:** qPCR Primers for *CSP12* of *O. d. asiaticus*.

Gene Name	Forward Primer (5′ to 3′)	Reverse Primer (5′ to 3′)
*OasiCSP12*	GCTACGCCAAGTACGACCAC	TCATGAAGTCCACCACCTTG
*β-actin*	CTACCACAGCCGAGCGAGAA	CCATCAGGCAGCTCGAAGGA

**Table 2:** Fluorescent competitive binding affinities of OasiCSP12 from *O. d. asiaticus* with different ligands.

Ligands Name	CAS	OasiCSP12	
		IC50 (μmol L^−1^)	Ki (μmol L^−1^)
Heptaldehyde	111-71-7	11.3	9.72
4-Ethyl-benzaldehyde	4748-78-1	12.07	10.39
2-Ethyl-1-hexanol	104-76-7	14	12.04
1-Ethyl-2-pyrrolidinone	2687-91-4	14.24	12.25
Isophthalaldehyde	626-19-7	14.88	12.8
4-Methoxystyrene	637-69-4	16.28	14.01
Dimethylphthalate	131-11-3	17.43	15
Diacetyl	431-03-8	18.88	16.25
Methacrolein	78-85-3	19.46	16.74
1,4-Diethyl-benzene	105-05-5	19.71	16.96
Dodecamethyl-cyclohexasiloxane	540-97-6	19.75	17
Phenylacetonitrile	140-29-4	19.98	17.19
2-Methylbutanal	96-17-3	25.06	21.57
Octanal	124-13-0	25.81	21.53
3-Ethyl-benzaldehyde	34246-54-3	26.88	23.13
Acetophenone	98-86-2	>30	—
Decamethyl-cyclopentasiloxane	541-02-6	>30	—
Dodecane	112-40-3	>30	—
1H-Indol-4-ol	2380-94-1	>30	—
Hydroxyacetone	116-09-6	>30	—
Nonanal	124-19-6	>30	—

**Table 3:** Main residue-level binding free energy and related component parameters of binding between *OasiCSP12* and 2-Methylbutanal, Phenylacetonitrile, Nonanal.

Ligand	Res	Binding (DH_Corr)	MM (DH_Corr)	PBSA	VDW	ELE (DH_Corr)	PB	SA
2-Methylbutanal	86VAL	−3.527	−2.623	−0.904	−2.602	−0.022	−0.427	−0.477
	71LEU	−3.116	−3.106	−0.01	−3.176	0.07	0.212	−0.222
	8LEU	−2.284	−4.173	1.89	−4.039	−0.134	2.238	−0.349
	90VAL	−2.137	−2.116	−0.022	−1.923	−0.193	0.311	−0.333
	101TRP	−1.776	−2.12	0.344	−2.091	−0.029	0.59	−0.245
phenylacetonitrile	71LEU	−7.271	−6.623	−0.649	−6.662	0.039	−0.121	−0.528
	118ILE	−5.015	−5.348	0.333	−4.658	−0.69	0.918	−0.584
	90VAL	−3.42	−3.265	−0.155	−3.231	−0.035	0.201	−0.356
	101TRP	−2.564	−4.479	1.915	−4.548	0.069	2.501	−0.586
	108TYR	−2.321	−2.705	0.385	−2.497	−0.208	0.539	−0.154
Nonanal	63LEU	−4.983	−4.354	−0.629	−4.452	0.098	0.038	−0.667
	60ILE	−4.157	−3.86	−0.297	−3.855	−0.005	0.18	−0.478
	28TYR	−3.69	−7.242	3.552	−6.353	−0.889	4.521	−0.969
	89VAL	−3.085	−2.712	−0.373	−2.688	−0.024	−0.068	−0.305
	33VAL	−2.623	−2.452	−0.171	−2.35	−0.102	0.048	−0.219
Heptaldehyde	118ILE	−4.62	−4.685	0.065	−4.769	0.084	0.532	−0.466
	104LEU	−4.512	−4.31	−0.202	−4.717	0.407	0.082	−0.285
	101TRP	−4.418	−8.911	4.493	−6.983	−1.928	5.211	−0.717
	71LEU	−3.595	−3.297	−0.298	−3.298	0.001	−0.064	−0.235
	15LEU	−2.593	−2.091	−0.502	−2.172	0.081	−0.233	−0.27

Notes: All energy values are reported in kcal·mol−1. Binding free energy and its decomposition components were calculated using the MM/GBSA (Molecular Mechanics/Generalized Born Surface Area) method with hydrogen bond correction. Res: Amino acid residue involved in ligand binding; Binding(DH_Corr): Binding free energy with hydrogen bond correction; MM(DH_Corr): Molecular mechanics energy (encompassing van der Waals and electrostatic interactions) with desolvation and hydrogen bond correction; PBSA: Polar solvation energy calculated via the Poisson-Boltzmann Surface Area model; VDW: Van der Waals energy component; ELE(DH_Corr): Electrostatic energy component with desolvation and hydrogen bond correction; PB: Polar solvation energy (Poisson-Boltzmann term); SA: Non-polar solvation energy derived from solvent-accessible surface area.

## Data Availability

The original contributions presented in this study are included in the article. Further inquiries can be directed to the corresponding authors.
